# Phenotypic and Genotypic Detection of Biofilm-Forming *Staphylococcus aureus* from Different Food Sources in Bangladesh

**DOI:** 10.3390/biology11070949

**Published:** 2022-06-22

**Authors:** Fatimah Muhammad Ballah, Md. Saiful Islam, Md. Liton Rana, Farhana Binte Ferdous, Rokeya Ahmed, Pritom Kumar Pramanik, Jarna Karmoker, Samina Ievy, Md. Abdus Sobur, Mahbubul Pratik Siddique, Mst. Minara Khatun, Marzia Rahman, Md. Tanvir Rahman

**Affiliations:** Department of Microbiology and Hygiene, Faculty of Veterinary Science, Bangladesh Agricultural University, Mymensingh 2202, Bangladesh; moncher.ballah13@gmail.com (F.M.B.); dvm41257@bau.edu.bd (M.S.I.); liton.21110215@bau.edu.bd (M.L.R.); farhanaferdous1501184@gmail.com (F.B.F.); rokeya44314@bau.edu.bd (R.A.); pritom46952@bau.edu.bd (P.K.P.); jarna.20210210@bau.edu.bd (J.K.); v.samina@gmail.com (S.I.); soburvetbau@gmail.com (M.A.S.); mpsiddique@bau.edu.bd (M.P.S.); mmkhatun@bau.edu.bd (M.M.K.); marzia_micro@bau.edu.bd (M.R.)

**Keywords:** biofilm formation, *S. aureus*, CRA plating test, CVMP test, *ica* genes, *bap* gene, foods, public health, Bangladesh

## Abstract

**Simple Summary:**

Biofilm formation by *Staphylococcus aureus* in foods poses a potential concern for public health and food safety. Therefore, the present study was conducted to detect biofilm-producing *S. aureus* from foods and human hand swabs using phenotypic and genotypic assays. In this study, *S. aureus* was detected in 23.81% (100/420) of samples, and among them, 89 and 97 of the isolates were biofilm producers by qualitative and quantitative tests, respectively. At least one biofilm-forming gene was detected in 21 *S. aureus* isolates, of which four isolates harbored all five adhesion genes (*icaA*, *icaB*, *icaC*, *icaD*, and *bap*). In addition, the occurrence of adhesion genes in *S. aureus* isolates showed a strong significant correlation among themselves. This is the first report on detecting biofilm-forming *S. aureus* from foods and hand swabs in Bangladesh using the molecular technique. The findings from this study indicate a significant public health risk and suggest the necessity of maintaining food hygiene practices at every step of the food chain to prevent and control *S. aureus* foodborne illness.

**Abstract:**

*Staphylococcus aureus* is a major foodborne pathogen. The ability of *S. aureus* to produce biofilm is a significant virulence factor, triggering its persistence in hostile environments. In this study, we screened a total of 420 different food samples and human hand swabs to detect *S. aureus* and to determine their biofilm formation ability. Samples analyzed were meat, milk, eggs, fish, fast foods, and hand swabs. *S. aureus* were detected by culturing, staining, biochemical, and PCR. Biofilm formation ability was determined by Congo Red Agar (CRA) plate and Crystal Violet Microtiter Plate (CVMP) tests. The *icaA*, *icaB*, *icaC*, *icaD*, and *bap* genes involved in the synthesis of biofilm-forming intracellular adhesion compounds were detected by PCR. About 23.81% (100/420; 95% CI: 14.17–29.98%) of the samples harbored *S. aureus*, as revealed by detection of the *nuc* gene. The CRA plate test revealed 20% of *S. aureus* isolates as strong biofilm producers and 69% and 11% as intermediate and non-biofilm producers, respectively. By the CVMP staining method, 20%, 77%, and 3% of the isolates were found to be strong, intermediate, and non-biofilm producers. Furthermore, 21% of *S. aureus* isolates carried at least one biofilm-forming gene, where *icaA*, *icaB*, *icaC*, *icaD*, and *bap* genes were detected in 15%, 20%, 7%, 20%, and 10% of the *S. aureus* isolates, respectively. Bivariate analysis showed highly significant correlations (*p* < 0.001) between any of the two adhesion genes of *S. aureus* isolates. To the best of our knowledge, this is the first study in Bangladesh describing the detection of biofilm-forming *S. aureus* from foods and hand swabs using molecular-based evidence. Our findings suggest that food samples should be deemed a potential reservoir of biofilm-forming *S. aureus*, which indicates a potential public health significance.

## 1. Introduction

*Staphylococcus aureus* is a food-borne pathogen ranked as the third most common bacterial cause of foodborne illness worldwide [1]. Consuming foods contaminated with heat-stable staphylococcal enterotoxins is a major cause of staphylococcal food poisoning, which leads to abdominal cramps, diarrhea, nausea, vomiting, endocarditis, pneumonia, toxic shock, and even skin infections in humans [2]. Food handlers, hand contact surfaces, and food contact surfaces are important sources of *S. aureus* transmission in food processing facilities, particularly during food processing and packaging [3].

Various pathogenic bacteria or other spoilage bacteria can be attached to hand and food contact surfaces as planktonic or adherent cells to form a biofilm. Biofilms are a common approach adopted by bacteria to endure various hostile environmental conditions by forming an aggregation of microbial cells surrounded by exopolymeric substances [4]. Biofilm-forming bacteria have several advantages over planktonic cells by showing more resistance to environmental stress conditions, sanitizers, and antimicrobials. Bacterial attachment and biofilm formation are affected by factors such as bacterial types, contact surface characteristics, growth conditions, and other environmental factors [5].

*Staphylococcus aureus* affects food quality and safety by persisting and developing biofilms in food processing environments. Biofilm development in *S. aureus* depends on five stages: initial attachment, unchangeable attachment, first maturation, second maturation, and finally detachment [6]. The extracellular matrix of staphylococcal biofilms includes exopolysaccharide, proteaceous, and extracellular DNA [7]. Exopolysaccharide, also termed Polysaccharide Intercellular Adhesin (PIA) or Poly-β-1,6-N-acetyl-D-glucosamine (PNAG), is the first widely researched matrix element. Its production and secretion are generated by a protein expressed in the *icaADBC*, an intercellular adhesion (*ica*) operon that includes *icaA* and *icaB* (both belong to N-acteylglucosamine transferase), *icaC* (belongs to an anticipated exporter), and *icaD* (belongs to a deacetylase) [8]. The staphylococcal biofilm formation significantly depends on the *ica* operon and environmental factors such as temperature, osmotic pressure, glucose, and low oxygen to induce its expression [9]. Certain species of *S. aureus* may also encode a microbial surface component named biofilm-associated protein (*bap*) that recognizes adhesive matrix substances and confers PIA production and biofilm development independently via cell-to-cell aggregation [10]. These *bap* and *bap*-associated proteins have the ability to be present on bacterial surfaces, show virulence properties, and control mobile elements [11].

Contamination of biofilm-forming *S. aureus* in food sources is a serious public health concern. Understanding staphylococcal biofilm formation is, therefore, pivotal to developing strategies for preventing biofilm-related contamination. As we know, there is no report on detecting biofilm-forming *S. aureus* phenotypically and genotypically from food sources in Bangladesh. The present study was therefore aimed: (1) to investigate biofilm production in *S. aureus* isolates by phenotypic and quantification approaches and (2) to evaluate different biofilm-related genes (*icaADBC* and *bap*) of *S. aureus* isolated from different food sources in Bangladesh.

## 2. Materials and Methods

### 2.1. Sample Size Calculation

Because no previous research had been conducted in Bangladesh, we could estimate the prevalence of biofilm-forming *S. aureus* to be 50%. Therefore, the sample size was enumerated following the formula described by Thrusfeld [12]:

$$n=\frac{Z^{2}pq}{d^{2}}$$

where *n* = desired sample size, Z = the standard normal deviation at 95% confidence level (1.96), *p* = assumed prevalence (50% = 0.5), q = 1 − *p* = (1 − 0.5) = 0.5, d = precision (it may be 5% or 10%, for the best accuracy we assume 5%, so d = 0.05). Therefore, *n* = (1.96)^2^ × 0.5 × 0.5/(0.05)^2^ = 384. To account for non-response, 10% more samples were calculated, and then the sample size was = (384 + 10% of 384) = (384 + 38.4) ≈ 422. However, 420 samples were collected related to foods and food products.

### 2.2. Study Area and Sampling

**Figure 1:**
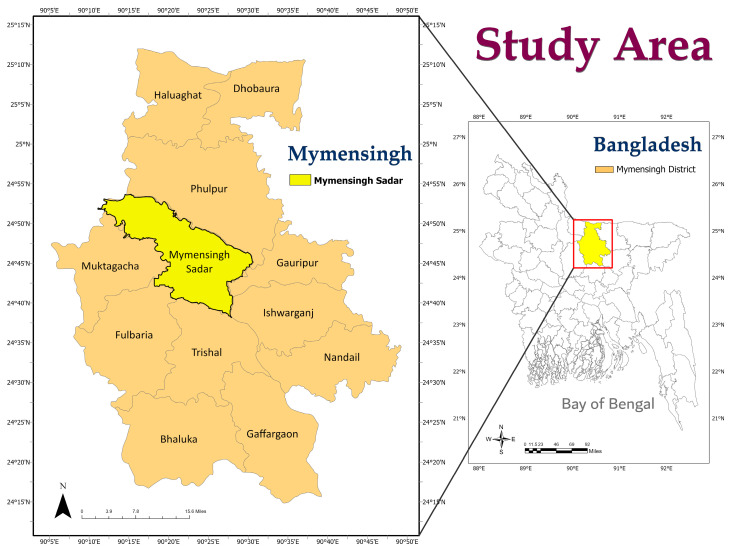
Sample location map of the study area. The map was created by ArcMap 10.7 (ArcGIS Enterprise, ESRI, Redlands, CA, USA).

The present study was carried out in Mymensingh Sadar Upazila (24.7851° N, 90.3560° E), Mymensingh district of Bangladesh, between June 2021 and March 2022. The study area is featured in Figure 1.

A total of 420 samples associated with food and food products were collected aseptically, comprising human hand swab—60, raw milk—60, chicken muscle—60 (breast—30 and thigh—30), fish—60, egg surface—60, ready-to-eat foods—120 (fuchka—20, French fries—20, vegetable fries—20, puri—20, singara—20, and samosa—20).

Hand swabs were taken from vendors and dairy farms’ owners using sterile cotton buds, followed by immediately transferring into sterile test tubes containing 5 mL of nutrient broth (NB; HiMedia, India); 4 mL of raw milk (immediately after milking) was collected by sterile falcon tubes from different dairy farms; breast (25 g) and thigh (25 g) muscles from each chicken were collected by sterile zip-lock bags from poultry slaughterhouses; fresh fishes were collected from different local fish markets and eggs from egg markets using sterile zip-lock bags. In addition, ready-to-eat foods were collected from different food vendors and restaurants using sterile zip-lock bags. After collecting samples, particular tag numbers were given and transferred to the laboratory by maintaining a cold chain.

### 2.3. Sample Processing

After taking samples to the laboratory, each raw milk sample collected was vortexed, and 1 mL of sample was transferred into 5 mL of NB, contained in a sterile test tube. For chicken meat, 5 g of each sample was ground and transferred into 5 mL of NB, contained in a sterile test tube, followed by mixing homogenously. For ready-to-eat food samples, 20 mL of sterile phosphate buffer saline (PBS) was added to each sample and mixed by grinding with a sterile mortar and pestle. The mixture was then centrifuged at 4000 rotations per minute (rpm) for seven minutes in a centrifuge machine (KUBOTA 6500, Japan), followed by the collection of 100 µL of supernatant and transfer into 5 mL of sterile NB, contained in a sterile test tube. For egg samples, the surface of each egg was washed thoroughly using 5 mL of sterile PBS. Subsequently, 50 µL of egg-washed samples were taken into sterile test tubes containing 5 mL of NB. For fish samples, the gills were collected by an expert veterinarian, and swabs of the gills using sterile cotton buds were taken and transferred into 5 mL of sterile NB, contained in a sterile test tube. After transferring all samples into the sterile test tubes, they were incubated aerobically in an incubator overnight at 37 °C for microbial enrichment.

### 2.4. Isolation of S. aureus

*S. aureus* were initially isolated by culturing on Mannitol Salt Agar (MSA) (HiMedia, India) media. At first, one loopful of overnight-cultured broth was streaked on separate MSA plates and subsequently incubated aerobically under the appropriate time (24 h) and temperature (37 °C). Isolates having golden-yellow colonies on MSA agar plates were assumed to be *S. aureus*, and they were sub-cultured on MSA plates to obtain pure colonies. Moreover, the purity of the colonies was checked by culturing on 5% bovine blood agar plates, followed by incubating at 37 °C for 24 h. Presumptive *S. aureus* colonies were then isolated by Gram staining, Voges–Proskauer tests, glucose and mannitol utilization tests, catalase tests, and coagulase tests [13].

### 2.5. Molecular Detection of S. aureus

Presumptive staphylococcal isolates were subjected to polymerase chain reaction (PCR) to detect *S. aureus* molecularly by targeting the gene *nuc* (Table 1).

For the molecular detection of *S. aureus*, DNA was extracted by the boiling method, as previously described [19,20]. In brief, first, 1 mL of overnight growth *S. aureus* culture was centrifuged at 5000 rpm for 5 min in a centrifuge machine (KUBOTA 6500, Kubota Corporation, Tokyo, Japan). The supernatant was then discarded, and a similar process was followed by adding 1 mL of PBS. The supernatant was discarded again, and the remaining pellet was suspended with 200 µL of PBS. The suspension was then boiled and cooled for 10 min in each step before being centrifuged at 10,000 rpm for 10 min in a centrifuge machine (KUBOTA 6500, Kubota Corporation, Tokyo, Japan). In the final step, the supernatant was collected as genomic DNA and stored at −20 °C for future studies.

All the PCRs were performed with a final volume of 20 µL (nuclease-free water- 4 µL, master mix (2x, Promega, Madison, WI, USA)- 10 µL, forward and reverse primers- 1 µL each, genomic DNA- 4 µL). After the completion of amplification, the PCR products were electrophoresed on a 1.5% agarose gel, subsequently stained with ethidium bromide, and recorded on a UV transilluminator (Biometra, Göttingen, Germany). Note that a 100 bp (Promega) DNA ladder was used to check the expected band size of the amplified PCR products.

### 2.6. Biofilm Formation of S. aureus

#### 2.6.1. Phenotypic Analysis of Biofilm Formation

Biofilm formation of *S. aureus* was phenotypically analyzed by the Congo Red (CR) test, as previously described [21]. In the CR test, the production of biofilm in the strains of *S. aureus* was studied by culturing the isolates on Congo Red Agar (CRA) plates. To prepare CRA plates, 0.8 g CR (HiMedia, India) and 36 g saccharose (HiMedia, India) were added to 1000 mL of blood agar (HiMedia, India) and subsequently incubated overnight at 37 °C to check their sterility. Then, overnight-growth *S. aureus* cultures were streaked on CRA plates, followed by incubation for 24 h and 48 h at 37 °C. The observable properties of the examined isolates were analyzed to check their biofilm-forming abilities. Isolates showing dry filamentous crusty black, pink with a dark center, and smooth pink colonies were interpreted as strong, intermediate/potential, and non-biofilm producers, respectively [22].

#### 2.6.2. Quantitative Analysis of Biofilm Formation

The biofilm-forming ability of *S. aureus* was measured using 96-well flat-bottomed microtiter polystyrene plates, as previously described by Kouidhi et al. [23]. Briefly, single colonies from CRA plates were inoculated into 5 mL of sterile tryptic soy broth (TSB), followed by incubation at 37 °C for 18 h without shaking. The growth of the isolates was adjusted with the 0.5 McFarland concentration, corresponding to a cell concentration of approximately 10^8^ colon-forming units (CFU)/mL for each strain [3]. The growth cultures were then diluted by a 10-fold dilution method in TSB supplemented with 10% glucose. An amount of 200 µL of the diluted culture was dispensed in three wells of the microtiter plate for each strain and incubated at 37 °C for 24 h. Negative controls were wells filled with broth medium (TSB + 10% glucose). Planktonic bacteria/cells were removed by washing each microtiter well 3–5 times with sterile PBS. The adherent cells were fixed with ethanol (95%) for 5 min, followed by emptying and drying the plates and subsequent staining for a few minutes with 100 µL of 1% crystal violet. The plates were air-dried after rinsing off the excess stain using sterile distilled water. The optical density (OD) value was evaluated at 570 nm (OD_570_) in an automatic spectrophotometer (VWR, part of Avantor, Radnor, PA, USA). The biofilm formation assay of each isolate was graded as strong biofilm (OD_570_ ≥ 1), moderate/intermediate biofilm (0.1 ≤ OD_570_ < 1), and non-biofilm (OD_570_ < 0.1) producers [23].

#### 2.6.3. Genotypic Analysis of Biofilm Formation

The molecular detection of biofilm-forming *S. aureus* was performed by PCR-based amplification of adhesion genes of the *icaADBC* operon (*icaA*, *icaB*, *icaC*, and *icaD*) and biofilm-associated proteins (*bap* gene). Table 1 summarizes the primer sequences, the PCR product size, and the corresponding references. The PCR amplification of *icaADBC* genes in *S. aureus* was performed using the same method that was used to detect the *nuc* gene.

### 2.7. Statistical Analysis

Data obtained from the present study were entered into Excel 365 (Microsoft/Office 365, Redmond, DC, USA) and were subsequently transferred to the GraphPad Prism (Prism 8.4.2, San Diego, CA, USA) and the Statistical Package for Social Science (IBM SPSS 25.0, Chicago, IL, USA) for statistical analysis.

#### 2.7.1. Descriptive Analysis

The prevalence of different variables was enumerated by descriptive analysis. The Wilson and Brown Hybrid method [24] was employed to calculate the binomial 95% confidence intervals for estimating the prevalence of different variables related to *S. aureus*-positive isolates. In addition, the chi-square test for goodness-of-fit was employed to determine the difference in the prevalence of *S. aureus* among different foods and hand swab samples. A *p*-value less than 0.05 (*p* < 0.05) was fixed to consider them statistically significant outcomes.

#### 2.7.2. Bivariate Analysis

A bivariate analysis was undertaken to determine the correlation between pairs of different genes associated with biofilm-forming *S. aureus* isolates. The bivariate analysis to calculate Pearson correlation coefficients (ρ) was performed by the SPSS analysis tool. The correlation was statistically significant only when the *p*-value was less than 0.05 (*p* < 0.05).

#### 2.7.3. Heatmap Analysis

A heatmap was generated to visualize the occurrence of staphylococcal biofilm-producing genes. The Origin Pro-2019b (Version 9.65, OriginLab Corporation, Northampton, MA, USA) was used to develop the heatmap. We considered the value “1” as positive and the value “0” as negative on the origin datasheet.

## 3. Results

### 3.1. Prevalence of S. aureus Isolates

Out of 420 samples analyzed, 151 (35.95%, 95% CI: 31.51–40.65%) samples were positive for *S. aureus* by observing the characteristic colonies of *S. aureus* on MS agar plates, Gram-staining, and biochemical tests. By PCR assay, 100 (23.81%; 95% CI: 19.99–28.11%) were found to be positive for *S. aureus*, as revealed by the detection of the *nuc* gene. Among the positive isolates, the highest prevalence was observed in raw milk, chicken muscle, fish, and egg surface samples, which showed equal prevalence (25%). Conversely, ready-to-eat food samples showed the lowest prevalence (21.67%). However, there was no statistically significant variation (*p* > 0.05) in the prevalence of *S. aureus* isolated from different food sources (Table 2).

### 3.2. Phenotypic Biofilm Formation

**Figure 2:**
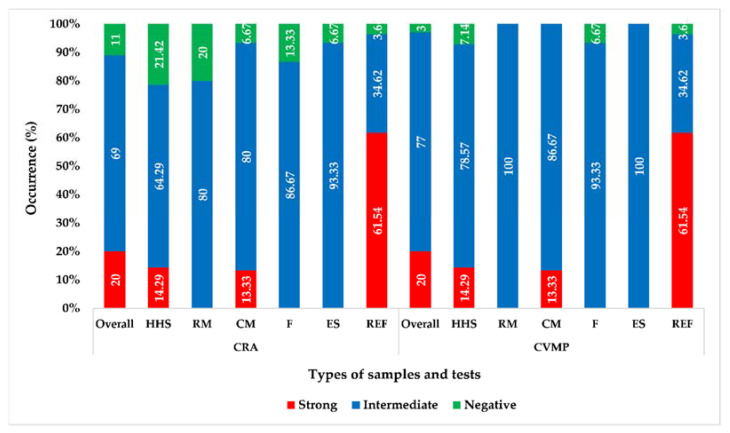
Occurrence of biofilm-producing *S. aureus* isolated from different foods and hand swabs, CRA = Congo Red Agar plating test, CVMP = Crystal Violet Microtiter Plate test, HHS = Human Hand Swab, RM = Raw Milk, CM = Chicken Muscle, F = Fish, ES = Egg Surface, REF = Ready-to-eat Food.

On the CRA plates, 20% (20/100, 95% CI: 13.34–28.88%) of *S. aureus* isolates were strong biofilm producers, while 69% (69/100, 95% CI: 59.37–77.22%) and 11% (11/100, 95% CI: 6.25–18.63%) isolates were intermediate and non-biofilm producers, respectively (Figure 2). Sample-wise, the highest occurrence of strong biofilm-producing *S. aureus* was found in ready-to-eat food samples (61.54%, 16/26), but there was no strong biofilm-producing *S. aureus* in raw milk, fish, or egg surface samples (Figure 2).

### 3.3. Quantification of Biofilm Formation

By the CVMP test, 20 (20%, 95% CI: 13.34–28.88%), 77 (77%, 95% CI: 67.85–84.16%), and 3 (3%, 95% CI: 0.82–8.45%) of *S. aureus* isolates showed strong, intermediate, or non-biofilm-producing characteristics, respectively (Figure 2). Like the CRA plate test, the CVMP test also revealed that ready-to-eat food samples exhibited the highest occurrence (61.54%, 16/26) of strong biofilm-producing *S. aureus* isolates, while raw milk, fish, and egg surface samples did not harbor any strong biofilm-producing *S. aureus* isolates (Figure 2). In addition, all the 89 biofilm-producing isolates detected by the CRA test were also detected as biofilm producers by the CVMP test.

### 3.4. Genotypic Biofilm Formation

**Figure 3:**
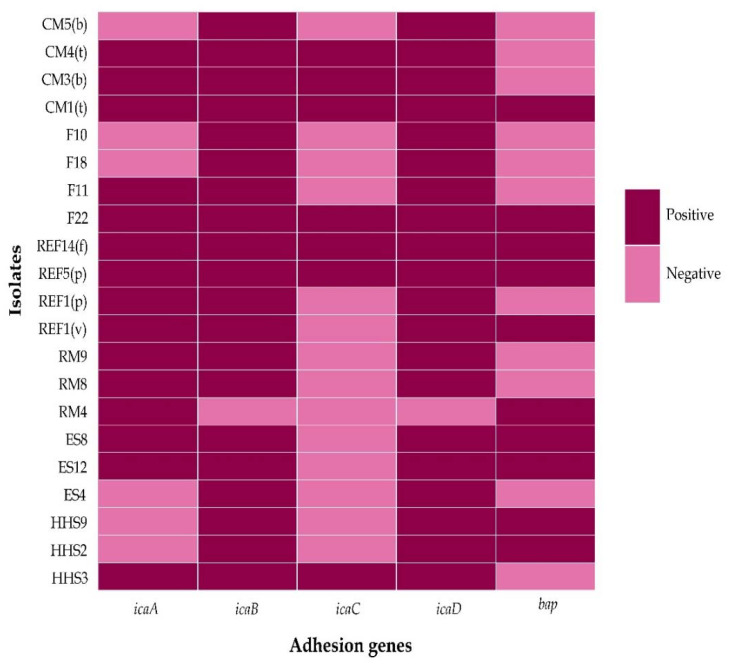
Heatmap showing the distribution of biofilm-forming genes in *S. aureus* isolated from different food samples and hand swabs, HHS = Human Hand Swab, ES = Egg Surface, RM = Raw Milk, REF = Ready-to-eat Food, v = Vegetable Fries, *p* = Puri, f = Fuchka, F = Fish, CM = Chicken Muscle, t = Thigh, b = Breast.

By PCR, out of 100 positive *S. aureus* isolates, 21 (21%, 95% CI: 14.17–29.98%) were found to carry at least one biofilm-forming gene. Among them, *icaB* and *icaD* were found to have the highest prevalence (20%, 95% CI: 13.34–28.89%) in the isolated *S. aureus* from different food samples and hand swabs, followed by *icaA* (15%, 95% CI: 9.31–23.28%), *bap* (10%, 95% CI: 5.52–17.44%), and *icaC* (7%, 95% CI: 3.43–13.75%) genes (Figure 3).

Out of 21 biofilm-forming *S. aureus* isolates, three and four adhesion genes were present in six isolates, two genes in five isolates, and all the selected genes were detected in four *S. aureus* isolates (Figure 3).

By bivariate analysis, strong positive significant correlations were observed between the biofilm-forming genes *icaA* and *icaB* (ρ = 0.770, *p* < 0.001), *icaA* and *icaC* (ρ = 0.653, *p* < 0.001), *icaA* and *icaD* (ρ = 0.770, *p* < 0.001), *icaA* and *bap* (ρ = 0.607, *p* < 0.001), *icaB* and *icaC* (ρ = 0.549, *p* < 0.001), *icaB* and *icaD* (ρ = 1.000, *p* < 0.001), *icaB* and *bap* (ρ = 0.583, *p* < 0.001), *icaC* and *icaD* (ρ = 0.549, *p* < 0.001), *icaC* and *bap* (ρ = 0.431, *p* < 0.001), *icaD* and *bap* (ρ = 0.583, *p* < 0.001) (Table 3).

## 4. Discussion

*Staphylococcus aureus* is a zoonotic pathogen that can cause a wide range of symptoms in humans [25]. The ability of *S. aureus* to form biofilm on food contact surfaces is considered to lead to food poisoning. Biofilm-producing *S. aureus* poses a serious health problem to humans. As we know, there is no previous report on the prevalence of biofilm-forming *S. aureus* from various food samples and hand swabs in Bangladesh.

In the current study, 23.81% of the food samples and hand swabs were found to be positive for *S. aureus* by the presence of the *nuc* gene. Previously in Bangladesh, Islam et al. [26] reported a similar occurrence of *S. aureus* (22%) in ready-to-eat foods, raw meat, raw milk, and fish samples, and a lower occurrence of *S. aureus* (6.67%) was detected in food samples by Urmi et al. [2]. In addition, Jahan et al. [27] and Pandit et al. [28] also detected *S. aureus* in raw milk (12/47) and on chicken egg surfaces (27/300) samples in Bangladesh. In addition, several previous studies showed diversified detection rates of *S. aureus* in different food samples abroad [29,30,31,32,33,34]. The observed variations between these studies and the present study could be attributed to differences in sample sources, size, and types; geographical locations; hygienic management; and other factors. Furthermore, ready-to-eat foods can be easily contaminated with *S. aureus* by food handlers, as most of them process and serve street foods with their bare hands in Bangladesh. In addition, proper hygiene practices are not guaranteed during food preparation; 23.33% of food-handler swab samples were found positive for *S. aureus*. Although there is no report in Bangladesh on the occurrence of *S. aureus* in food handlers’ hand swabs, Gadaga et al. [35] from Zimbabwe and Kasturwar and Shafee [36] from India recorded *S. aureus* in 32% and 36%, respectively, of samples from food handlers responsible for the food processing and serving. However, the presence of *S. aureus* in different food samples and hand swabs indicates a high health risk to consumers. In addition, the contamination level of food sources with *S. aureus* suggests that animal handling, food processing and handling, as well as cleaning and disinfecting food environments, must be improved.

The biofilm or slime formation on media is associated with the development of extracellular polysaccharides, which plays a significant role in bacterial adhesion [37,38]. Although the CRA test is not considered the most sensitive for determining biofilm development, this simple qualitative phenotypic test was used in this study because of its acceptable sensitivity and specificity [39]. In the present study, among 100 *S. aureus* isolates, 89% of the isolates showed characteristic colonies on CRA and were categorized as biofilm producers. The CVMP test revealed that 97 (97%) of the *S. aureus* isolates had the ability to produce biofilm. In addition, the same 20 *S. aureus* isolates were categorized as strong biofilm producers by both qualitative phenotypic and quantitative biofilm assays. The high and similar percentage of biofilm producers was in accordance with the previous study [10], which reported that 75% (63/84) and 97.62% (82/84) of the *S. aureus* isolated from food contact surfaces were biofilm producers by qualitative (CRA) and quantitative (crystal violet staining) assays, respectively. The variation in glucose concentration in the media used in the current study could explain the disparity in the results of the two tests. Rohde et al. [15] stated that adding 1% glucose to the TSB media could enhance the occurrence of biofilm formation in the *S. aureus* isolates by up to 83%. A previous study [40] reported that the expression of biofilm formation in *S. epidermis* isolates on CRA preparations with different amounts of glucose was significantly higher when the glucose concentration was higher.

This study recorded that 21% (21/100) of the *S. aureus* isolates harbored at least one biofilm-producing gene involved in the synthesis of PIA, where there was no difference in the distribution patterns of the *icaB* and *icaD* genes in the biofilm-forming *S. aureus* isolates. Chen et al. [3] also reported that the distribution of *icaB* and *icaD* genes was similar; however, the *bap* gene was not detected in the *S. aureus* isolates. In addition, the prevalence rates of other biofilm-related genes observed in the current study varied greatly, which can be supported by different studies [41,42,43]. Multifarious gene expression patterns can be recorded when *S. aureus* isolates are subjected to various temperatures and contact surfaces for varying lengths of time [3].

The present study revealed that the occurrence rates of biofilm-producing genes are much lower than the results observed in the CRA and CVMP tests. Similar findings were reported by previous studies [44,45], however, the high and similar prevalence rates of biofilm-producing *S. aureus* observed in the qualitative, quantitative, and molecular assays were recorded in previous studies [10,46,47]. This discrepancy could be explained by the different factors that are associated with biofilm formation in the *S. aureus* isolates. The interaction among different regulatory systems and the expression among different adhesion genes regulate biofilm formation, which can be triggered by various environmental factors such as the concentration of glucose in the used media, the temperature of the contact surfaces, the osmolality of the used media, and the growth conditions of the organisms [38,48,49]. In addition, the phenotypic characteristics of biofilm formation can be influenced by a discrepancy in the regulation of locus genes’ and putative adhesion genes’ expression [50]. Other credible mechanisms for the emergence of biofilm-negative strains of *S. aureus* include the disruption of genes by insertional inactivation and point mutations in the locus of adhesion genes [51]. The high variability of biofilm formation observed in the present study indicates that the determination of genes involved in the PIA or PNAG is not a complete determining factor of *S. aureus*’s ability to produce biofilm. Therefore, an integration of both phenotypic and genotypic tests could be used to identify biofilm-producing *S. aureus* isolates more accurately.

## 5. Conclusions

As we know, this is the first study in Bangladesh to detect biofilm-producing *S. aureus* phenotypically and genotypically from food. This study revealed that food samples and hand surfaces are prone to contamination with *S. aureus*, and most of the isolates could produce biofilm. Biofilm is a significant virulent substance in *S. aureus* infections, making their eradication difficult. The presence of biofilm-forming *S. aureus* in food samples and hand swabs indicates that food sources could act as a potential reservoir for pathogenic bacteria, posing serious public health significance. Our results are encouraging for further research focusing on the evaluation and exploration of the detailed genetic background of *S. aureus* isolated from food sources to assess their association with biofilm formation and infection development abilities. In addition, our findings emphasize the importance of good hygiene practices and installing strict food safety strategies at every stage of the food chain for preventing food-borne illnesses developed by *S. aureus* contamination and minimizing their cross-contamination hazards.

## Figures and Tables

**Table 1:** List of primers used in the present study to detect biofilm-producing *S. aureus* from food sources and human hand swabs.

Targeted Genes	Primer Sequence (5′–3′)	Annealing Temperature	Amplicon Size (bp)	References
*nuc*	F: GCGATTGATGGTGATACGGT R: AGCCAAGCCTTGACGAACTAAAGC	55	279	[14]
*icaA*	F: GACCTCGAAGTCAATAGAGGT R: CCCAGTATAACGTTGGATACC	56	814	[15]
*icaD*	F: AGGCAATATCCAACGGTAA R: GTCACGACCTTTCTTATATT	59	526	[16]
*icaB*	F: ATCGCTTAAAGCACACGACGC R: TATCGGCATCTGGTGTGACAG	59	526	[17]
*icaC*	F: ATAAACTTGAATTAGTGTATT R: ATATATAAAACTCTCTTAACA	45	989	[17]
*bap*	F: CCCTATATCGAAGGTGTAGAATTGCAC R: GCTGTTGAAGTTAATACTGTACCTGC	53	971	[18]

**Table 2:** Prevalence of *Staphylococcus aureus* isolated from different food sources and human hand swabs.

Name of Sample	Positive Isolates (%)	95% CI	*p*-Value
Human hand swab (*n* = 60)	14 (23.33)	14.44–35.44	0.992
Raw milk (*n* = 60)	15 (25)	15.78–37.23	
Chicken muscle (*n* = 60)	15 (25)	15.78–37.23	
Fish (*n* = 60)	15 (25)	15.78–37.23	
Egg surface (*n* = 60)	15 (25)	15.78–37.23	
Ready-to-eat foods (*n* = 120)	26 (21.67)	15.24–29.85	
Overall (*n* = 420)	100 (23.81)	19.99–28.11	

Here, a *p*-value less or equal to 0.05 (*p* ≤ 0.05) was statistically significant, *n* = Number of samples, CI = Confidence interval.

**Table 3:** Pearson correlation coefficients (ρ) for pairs of adhesion genes to assess biofilm-forming *S. aureus* isolates from different food samples and hand swabs (*n* = 100).

		*icaA*	*icaB*	*icaC*	*icaD*	*bap*
*icaA*	Pearson Correlation	1				
	Sig. (2-tailed)	-				
*icaB*	Pearson Correlation	0.770 **	1			
	Sig. (2-tailed)	0.000	-			
*icaC*	Pearson Correlation	0.653 **	0.549 **	1		
	Sig. (2-tailed)	0.000	0.000	-		
*icaD*	Pearson Correlation	0.770 **	1.000 **	0.549 **	1	
	Sig. (2-tailed)	0.000	0.000	0.000	-	
*bap*	Pearson Correlation	0.607 **	0.583 **	0.431 **	0.583 **	1
	Sig. (2-tailed)	0.000	0.000	0.000	0.000	-

Here, a *p*-value less than 0.05 (*p* < 0.05) was considered statistically significant, **. Correlation is significant at the 0.01 level (2-tailed), Sig. = Significance.

## Data Availability

Not applicable.
